# Eye Diseases

**Published:** 1874-02

**Authors:** 


### EYE DISEASES.

No one should ever make an application to the eye for any ailment whatever, stronger than luke warm water or milk, without the express directions of a physician. It is an organ so delicate in its nature and so easily destroyed that it should not be tampered with by what are called simple remedies. A lady was once advised to put a poultice of rotten apples on both her eyes one night for some weakness. She did so, and was blind next morning. There was no hope of restoration.

If anything serious is the matter with the eye apply to a physician or experienced occulist at once—men of standing in their profession, and who do not advertise in the newspapers. An aged lady suffered pain in the back part of the eye ball. The surgeon pronounced it a tumor, which would result fatally unless removed. The operation was performed by an eminent surgeon of this city, and, at the end of three years, the lady remains well at over three score and ten, although there is no sight in the eye—at least nothing distinct. We saw a piece of iron the other day, as large as the nail and joint of the thumb, which had been imbedded in the socket of the eye, and from which it had been removed by the tact and skill of an ophthalmic surgeon.

